# Investigating the role of *SORL1* in mediating AD‐specific endolysosomal phenotypes in neurons and microglia

**DOI:** 10.1002/alz.092619

**Published:** 2025-01-03

**Authors:** Brooke A. DeRosa, Yalun Zhang, Charles G. Golightly, Lauren Coombs, Lauren E. Rothschild, Brian W. Kunkle, Michael L. Cuccaro, Jeffery M. Vance, Margaret A Pericak‐Vance, Peter St George‐Hyslop, Derek M. Dykxhoorn

**Affiliations:** ^1^ John P. Hussman Institute for Human Genomics, University of Miami Miller School of Medicine, Miami, FL USA; ^2^ University of Toronto, Toronto, ON Canada; ^3^ John P. Hussman Institute for Human Genomics, University of Miami Miller School of Medicine, Miami, FL, USA, Miami, FL USA; ^4^ Dr. John T. Macdonald Foundation Department of Human Genetics, University of Miami Miller School of Medicine, Miami, FL USA; ^5^ Department of Neurology, University of Miami Miller School of Medicine, Miami, FL USA

## Abstract

**Background:**

Dysregulation of endolysosomal trafficking is a major pathogenic mechanism in Alzheimer’s disease (AD). From the family of AD‐linked endosomal pathway genes, *SORL1* stands out as one of the highest risk factors. *SORL1* encodes an endocytic sorting receptor that mediates endosomal trafficking and processing of key AD‐associated molecules, including pathogenic forms of amyloid‐β (e.g., Aβ42) and amyloid precursor protein (APP). Using complementary cell‐based model systems, we investigated the role of a protein‐truncating *SORL1* variant on endolysosomal trafficking and APP processing.

**Method:**

Induced pluripotent stem cell (iPSC) lines were derived from two siblings affected with early onset AD (EOAD) who carried a rare protein‐truncating deletion in *SORL1* (rs1343336951; p.C1431fs). *SORL1* ^+/+^ isogenic control iPSC lines were created from the patient lines using CRISPR/Cas9. After validation, the iPSC lines were differentiated into forebrain neurons and analyzed for endosomal trafficking defects. Additional analyses were completed in HEK293‐APPswe cells overexpressing SORL1 wild‐type (WT) or the C1431fs variant. Studies are underway that examine *SORL1* ^+/C1431fs^ microglia for defects in endolysosomal function.

**Result:**

*SORL1* ^+/C1431fs^ neurons have increased localization of APP in early endosomes (p = 0.002), endosomal swelling (p = 0.004), and greater numbers of early endosomes per cell (p = 0.018). We additionally observe *SORL1* ^+/C1431fs^ neurons trending toward increased secretion of Aβ42 compared to controls. In HEK293 cells, C1431fs increased secretion of Aβ42 (p < 0.01), Aβ40 (p < 0.01), as well as APP soluble α‐secretase (sAPPα; p < 0.01) and β‐secretase (sAPPβ; p < 0.01) cleavage products. We additionally found C1431fs led to increased secretion of soluble SORL1 (p < 0.01). Surface biotinylation revealed C1431fs led to lower levels of SORL1 detected at the cell surface (p < 0.05). Conversely, C1431fs increased the amount of surface APP (p < 0.05).

**Conclusion:**

Our results indicate that a single gene copy of the *SORL1* C1431fs variant is sufficient to induce neuronal defects in endosomal trafficking and APP processing. Our findings are consistent with previously reported results from similar studies of SORL1 in cultured neurons. Ongoing studies in *SORL1* ^+/C1431fs^ microglia and neurons will help broaden our understanding of the role *SORL1* plays in regulating endolysosomal phenotypes in multiple cell types implicated in AD.

